# Ubiquitin ligase RCHY1 regulates autophagosome-lysosome fusion

**DOI:** 10.1038/s41420-026-03088-w

**Published:** 2026-04-15

**Authors:** Ruchi Umargamwala, Jantina Manning, Julian M. Carosi, Donna Denton, Sharad Kumar

**Affiliations:** 1 Centre for Cancer Biology, University of South Australia, Adelaide, SA Australia; 2 Centre for Cancer Biology, College of Health, Adelaide University, Adelaide, SA Australia; 3 South Australian Health and Medical Research Institute, Adelaide, SA Australia

**Keywords:** Autophagy, Ubiquitin ligases

## Abstract

Autophagy is a fundamental cellular recycling process that maintains homeostasis during animal development and under nutrient-limiting conditions. In our previous work, we employed autophagy-dependent cell death (ADCD) in the obsolete *Drosophila* larval midgut as a model to identify the enzymes involved in protein modification via ubiquitination with potential roles in autophagy regulation. From a genetic screen we identified RING E3 ligase RCHY1 as a candidate regulator. Here, we demonstrate that RCHY1 is essential for autophagy regulation during larval midgut ADCD in *Drosophila* and promotes autophagic flux in HeLa cells. Loss of *Rchy1* impaired autophagosome–lysosome fusion and led to the accumulation of amphisomes in larval midgut cells. Similarly, depletion of RCHY1 in HeLa cells disrupted autophagic flux and reduced autolysosome formation, indicating evolutionary conservation of its function. Collectively, our findings identify RCHY1 as a putative regulator of autophagy that facilitates autophagosome-lysosome fusion.

## Introduction

Macroautophagy (hereafter referred to as autophagy) is a lysosome-mediated degradation pathway that recycles cytoplasmic cargo to sustain nutrient supply under adverse conditions, eliminate toxic or foreign substrates and preserve cellular homeostasis [1]. Cargo can be sequestered either randomly or selectively into double-membraned autophagosomes, which subsequently fuse with lysosomes where hydrolases drive degradation and recycling [2]. Perturbation of autophagy disrupts cell differentiation, metabolism and DNA repair pathways, contributing to detrimental health outcomes and accelerated ageing [3–5]. Thus, uncovering novel regulators of autophagy is important for identifying therapeutic targets that may be leveraged to promote cellular resilience and combat disease.

Precise regulation of autophagy is essential to ensure its timely activation and inhibition in response to cellular cues and this regulation is frequently mediated by post-translational modifications such as ubiquitination. The covalent attachment of ubiquitin to core autophagy-related proteins, orchestrated by E1, E2, and E3 enzymes, modulates autophagy at multiple stages, including initiation, autophagosome nucleation, autophagosome expansion and autophagosome-lysosome fusion [6]. For instance, K63-linked ubiquitination of ULK1 and Beclin-1 by the E3 ligase TNF receptor-associated factor 6 (TRAF6) promotes autophagy activation [7, 8]. In contrast, the E3 RING finger protein 2 (RNF2) mediates K48-linked proteasomal degradation of autophagy and Beclin-1 regulator 1 (AMBRA1), thereby suppressing autophagy [9]. Similarly, the E3 ligase SMAD ubiquitination regulatory factor 1 (SMURF1) enhances autophagosome maturation by attaching K29/K33 ubiquitin chains to UVRAG, disrupting its interaction with the suppressor Rubicon [10]. Conversely, the E3 NEDD4-like (NEDD4L) terminates autophagy under prolonged starvation by decorating ULK1 with K27 and K29 ubiquitin chains, leading to ULK1 degradation [11]. Numerous other E3 ligases have been implicated in fine-tuning autophagy through ubiquitination of autophagy machinery [6]. However, many ubiquitinated autophagy-related proteins remain without identified E1, E2, or E3 effectors, underscoring the need for further characterization of ubiquitination enzymes to uncover their roles in autophagy.

The steroid hormone 20-hydroxyecdysone (ecdysone) induces developmentally timed autophagy-dependent cell death (ADCD) in larval midgut cells by upregulating autophagy genes and suppressing prosurvival decapentaplegic (Dpp) signalling, to drive midgut histolysis [12–14]. This system provides a powerful model for RNAi-mediated knockdown of ubiquitination enzymes and subsequent characterization of candidate regulators of autophagy. In our previous work, we genetically screened more than 250 E1, E2 and E3 enzymes in *Drosophila melanogaster* larval midguts to uncover possible new roles for ubiquitination in autophagy regulation [15]. Here, we characterise one of the candidates identified in the screen, the E3 ligase RING finger and CHY zinc finger domain-containing protein 1 (RCHY1, also known as ARNIP, CHIMP, PIRH2 and RNF199) and demonstrate its putative function in regulating autophagosome-lysosome fusion.

## Results

### *Rchy1* depletion disrupts autophagy in *Drosophila* tissues

The insect larval midgut is comprised of four tissue appendages known as gastric caeca, the proventriculus, and midbody. In response to ecdysone signalling, ADCD of the midgut is initiated in late third instar larvae at −4 h relative to puparium formation (h RPF), resulting in degradation of gastric caeca appendages and proventriculus by +4 h RPF [13, 16].

**Fig. 1:**
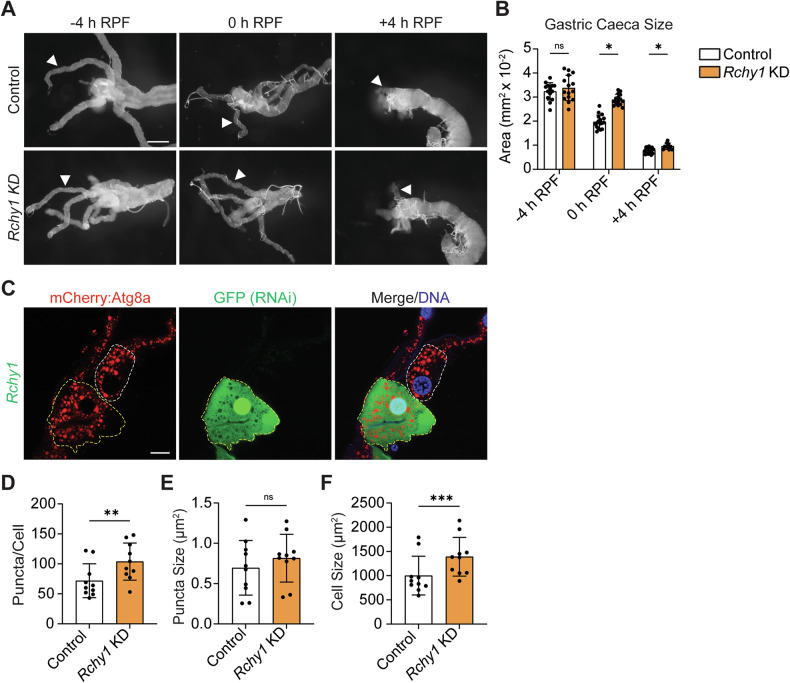
*Rchy1* KD disrupts autophagy in the *Drosophila* larval midgut. **A** *Rchy1* KD (*Mex-GAL4*/*UAS-Rchy1i*) midguts displayed significantly longer gastric caeca compared to controls (*Mex-GAL4*/*+*) at 0 h RPF and +4 h RPF. White closed arrows indicate gastric caeca. **B** Quantitation of gastric caeca length at -4 h RPF, 0 h RPF and +4 h RPF. **C** *Rchy1* KD (*hsFLP; p-mCherryAtg8a/UAS-Rchy1i; Act*>*CD2*>*GAL4, UAS-nlsGFP/+*) in GFP-labelled midgut cells (yellow dashed line) compared to adjacent GFP-negative control cells (white dashed line). Quantitation of **D** Atg8a-positive vesicle count and **E** puncta size and **F** cell size of control and *Rchy1* KD mosaic clones. Quantitation of Atg8a puncta represented as puncta/cell ± SD (paired t-test). Quantitation of Atg8a puncta size represented as µm^2^ ± SD (paired t-test). Quantitation of cell size represented as µm^2^ ± SD (paired t-test). Scale bar = 125 μm (**A**) and 10 µm (**C**). ns non-significant. *p* < 0.05 was considered statistically significant.

To determine if RCHY1 is important for autophagy regulation, we utilised the midgut-specific driver, *Mex*-GAL4, to perform RNAi-mediated knockdown (KD) of *Rchy1*. RNAi efficiency was assessed by qPCR (Supplementary Fig. 1). Larval midguts were collected at −4 h RPF, 0 h RPF and +4 h RPF for morphological analyses of gastric caeca during autophagy-dependent midgut histolysis. While *Rchy1* KD did not show a significant difference in gastric caeca size at −4 h RPF, later stages of degradation were significantly attenuated, resulting in consistently longer gastric caeca at all subsequent developmental timepoints (Fig. 1A, B). At +4 h RPF, where control midguts displayed small remnants of the obsolete appendages, gastric caeca in *Rchy1* KD midguts remained prominent, indicating that loss of *Rchy1* caused a significant delay in timely removal of the obsolete midgut (Fig. 1A, B). Additionally, we generated GFP-labelled *Rchy1* KD mosaic clones that expressed mCherry-tagged Atg8a (mCherry-Atg8a), a marker for autophagic compartments (Fig. 1C). *Rchy1* KD cell clones displayed a significant increase in the number of Atg8a-positive vesicles, although the size of these vesicles remained unchanged when compared to adjacent control cells (Fig. 1C–E). *Rchy1* KD mosaic clone cells were also significantly larger than GFP-negative cells, which suggests a relationship between autophagy and cell size reduction in larval midguts undergoing ADCD (Fig. 1F) [17]. Together, these data provide evidence that RCHY1 is important for timely degradation of the larval midgut by ADCD, and that this function is linked to Atg8a compartment dysfunction in larval midgut cells.

### RCHY1 facilitates autophagosome turnover in larval midgut cells

**Fig. 2:**
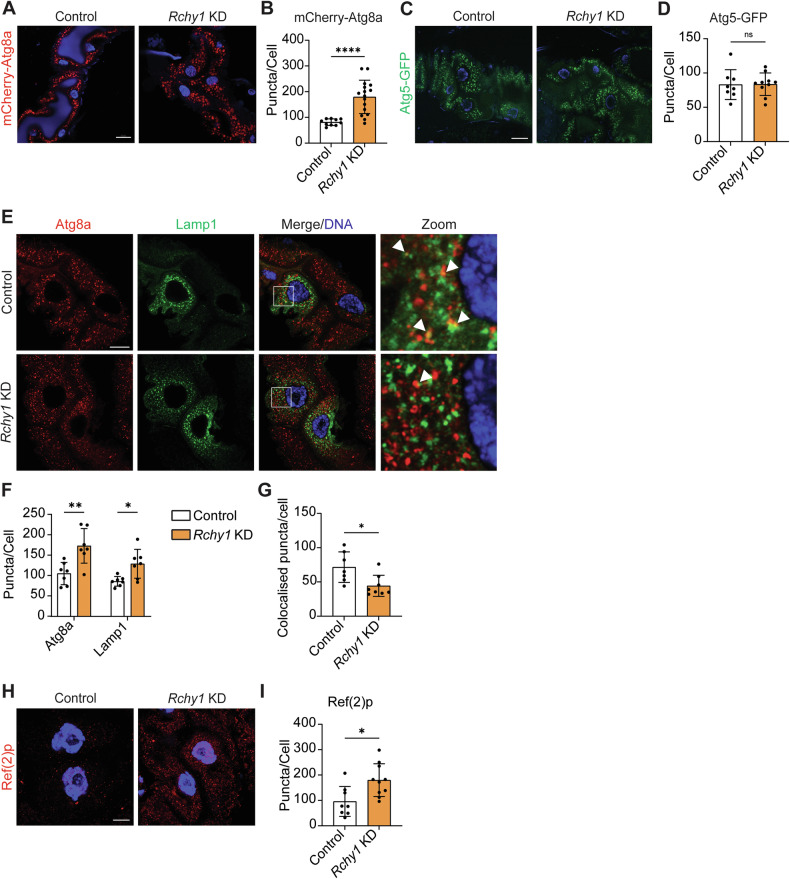
*Rchy1* KD results in reduced autophagosome turnover. **A** Live-imaging of midguts at -4 h RPF indicates an increase in mCherry-Atg8a-labelled autophagic structures upon *Rchy1* KD (*Mex-GAL4/UASRchy1i; p-mCherry-Atg8a/+*). **B** Quantitation of Atg8a-positive vesicles represented as average puncta/cell ± SD (two-tailed unpaired t-test). **C** Live-imaging of the phagophore-associated protein, GFP-ATG5, revealed no significant difference in levels of phagophores in *Rchy1* KD (*Mex-GAL4/UASRchy1i; UASeGFP-Atg5/+*) midguts at −4 h RPF. **D** Quantitation of Atg5-positive vesicles represented as average puncta/cell ± SD (two-tailed unpaired t-test). **E** Immunostain for autophagosomes (Atg8a, red) and lysosomes (Lamp1, green) in -4 h RPF *Rchy1* KD midguts (*Mex-GAL4, UAS-LAMP1-GFP/UASRchy1i*) compared to controls (*Mex-GAL4, UAS-LAMP1-GFP/+*). White squares show zoomed regions of interest. Colocalised puncta indicated with closed white arrows. **F** Quantitation of Atg8a-, Lamp1-, and **G** double-positive vesicles represented as average puncta/cell ± SD (two-tailed unpaired t-test). **H** Immunostaining for ref(2)p (red) at -4 h RPF revealed accumulation of ref(2)p puncta in *Rchy1* KD (*Mex-GAL4/UASRchy1i; UASeGFP-Atg5/+*) midguts. **I** Quantitation of ref(2)p puncta represented as average puncta/cell ± SD (two-tailed unpaired t-test). Scale bar = 20 µm (**A**, **C**), 10 µm (**E**, **H**). ns non-significant. *p* < 0.05 was considered statistically significant.

To further assess the role of RCHY1 in the regulation of Atg8a-positive vesicles, we performed live-imaging of larval midguts at −4 h RPF that were depleted of *Rchy1* using the midgut driver, *Mex-GAL4*, and endogenously expressed the autophagosome marker, mCherry-Atg8a. Similar to the effect of *Rchy1* KD in mosaic clones, we observed an increase in mCherry-Atg8a-positive vesicles (Fig. 2A, B). To determine if these vesicles were early autophagic structures (phagophores), we performed *Rchy1* KD in larval midguts expressing Atg5-GFP as a marker for phagophores. This revealed no significant difference in Atg5-positive vesicle numbers between control and *Rchy1* KD midguts, suggesting that the loss of *Rchy1* resulted in accumulation of mature autophagosomes rather than early autophagic vesicles (Fig. 2C, D).

We hypothesised that failure in autophagosome turnover may be the consequence of impaired autophagosome-lysosome fusion. To test this, we examined the interaction between autophagosomes and lysosomes in midguts by immunostaining for Atg8a and lysosomal associated membrane protein 1 (Lamp1), respectively. Upon *Rchy1* KD, there was a significant increase in Atg8a-positive autophagosomes and Lamp1-positive lysosomes at −4 h RPF in *Rchy1* KD midguts (Fig. 2E, F). However, there was a significant reduction in the numbers of colocalised Atg8a-positive and Lamp1-positive puncta which supports the notion that the increase in autophagosomes in *Rchy1* KD midguts occurred due to a reduction in autophagosome-lysosome fusion (Fig. 2G). We also noted that despite the abundance of Lamp1-positive vesicles in *Rchy1* KD midgut cells, there was a significant decrease in the acidification of these vesicles as indicated by LysoTracker staining (Supplementary Fig. 2A–C). We further assessed whether the loss of *Rchy1* affected recycling of the selective autophagy receptor (SAR), ref(2)P, due to impaired autophagosome turnover. As expected, ref(2)p puncta were significantly elevated in *Rchy1* KD midgut cells compared to controls (Fig. 2H, I). These data suggest that RCHY1 is required for autophagosome-lysosome fusion and lysosomal-mediated degradation of autophagic substrates.

### *Rchy1* depletion results in accumulation of amphisomes in larval midgut cells

**Fig. 3:**
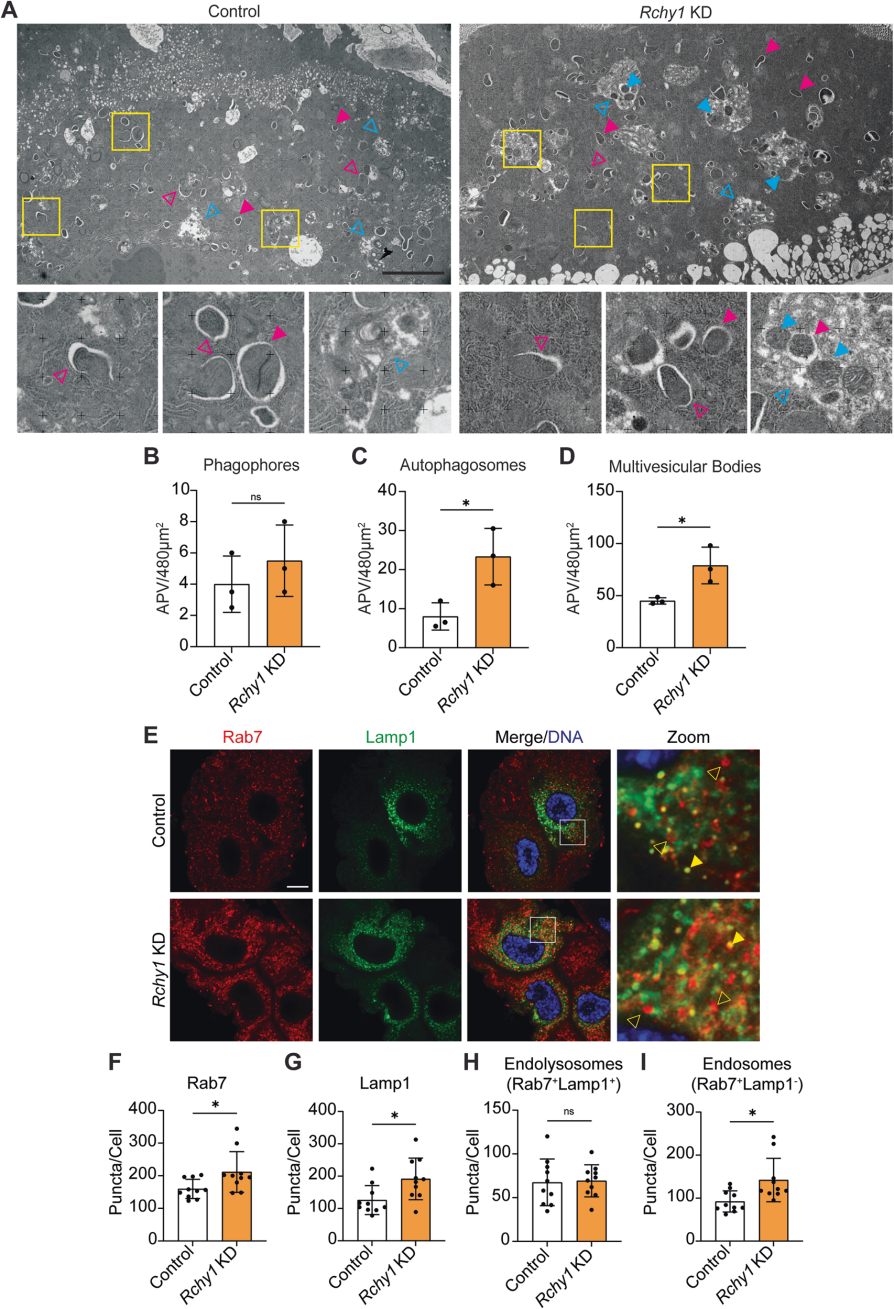
Loss of *Rchy1* causes accumulation of amphisomes and late endosomes in larval midgut cells. **A** Representative TEM micrographs of control (*Mex-GAL4*/+) and *Rchy1* KD (*Mex-GAL4*/*Rchy1i*) larval midguts at -4 h RPF. Quantitation of the number of **B** phagophores (open magenta arrows), **C** autophagosomes (closed magenta arrows), and **D** multivesicular bodies (MVBs, open cyan arrows) determined by point counting are represented as average points per vesicle (APV)/480µm^2^ ± SD (unpaired t-test). Yellow boxes denote zoomed regions of interest. Mitochondria within MVBs are indicated with closed cyan arrows. **E** Immunostain of −4 h RPF control (*Mex-GAL4*/+) and *Rchy1* KD (*Mex-GAL4*/*Rchy1i*) midguts for the late endosome/endolysosome marker, Rab7 (red), and lysosomal marker, Lamp1 (green). White boxes denote zoomed regions of interest. Quantitation of **F** Rab7, **G** Lamp1, **H** Rab7^+^LAMP1^+^ endolysosomes (closed yellow arrows) and (**I**) Rab7^+^Lamp1^−^ endosomes (open yellow arrows) represented as puncta/cell ± SD (unpaired t-test). Scale bar = 4 µm (**A**) and 10 µm (**E**). ns non-significant. *p* < 0.05 was considered statistically significant.

To validate the autophagy defects observed by fluorescence microscopy, we performed ultrastructural analysis of larval midguts using transmission electron microscopy (TEM). We noted a significant increase in autophagosomes (closed magenta arrows) in -4 h RPF *Rchy1* KD larval midgut cells that was consistent with live-imaging and immunostaining Atg8a data, although no changes in phagophore (open magenta arrows) numbers were observed (Fig. 3A–C). This also supported our earlier finding that loss of *Rchy1* did not change Atg5-GFP-positive vesicle numbers in larval midguts. Interestingly, loss of *Rchy1* led to the accumulation of large vesicles (open cyan arrows) that appeared to contain heterogenous cellular material (Fig. 3D). These have been characterised as multivesicular bodies (MVBs) due to their single limiting membrane that encloses multiple smaller intraluminal vesicles [18]. However, MVBs in *Rchy1* KD midguts also appeared to contain autophagosomes and mitochondria (closed cyan arrows), whereas these were not apparent in control midguts. This phenotype may be indicative of amphisome formation which occur when autophagosomes fuse with MVBs [19]. To investigate if *Rchy1* KD disrupts the endosomal pathway, we performed immunostaining on −4 h RPF control and *Rchy1* KD midguts for the late endosome/endolysosome marker, Rab7 and Lamp1 (Fig. 3E). The results show a significant increase in both Rab7- and Lamp1-positive vesicles, however, colocalisation did not significantly differ, indicating no disruption to endolysosome (Rab7^+^Lamp1^+^, closed yellow arrows) formation in *Rchy1* KD midgut cells (Fig. 3E–H). To determine if the increase in Rab7-positive vesicles was due to accumulation of endosomes, we subtracted Rab7^+^Lamp1^+^-positive puncta from the total Rab7 pool and found that Rab7^+^Lamp1^−^ (open yellow arrows) endosome numbers were significantly upregulated upon *Rchy1* loss (Fig. 3I). Together, these data suggest that *Rchy1* deficiency leads to formation of amphisomes in larval midgut cells.

### *Rchy1* genetically interacts with *Atg1*

**Fig. 4:**
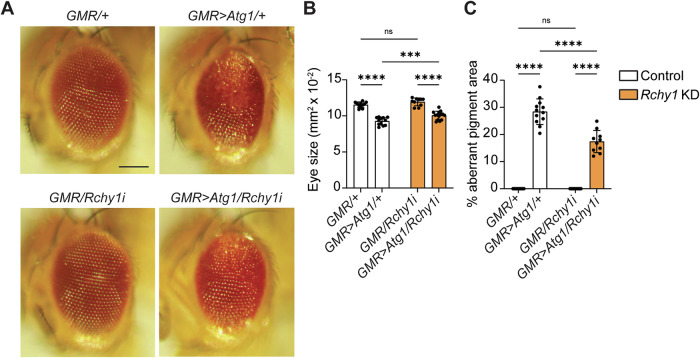
*Rchy1* KD rescues the *Atg1* rough eye phenotype in *Drosophila.* **A** Representative eye phenotypes for *Rchy1* RNAi crossed with *GMR-GAL4* (*GMR-GAL4/Rchy1i*) and *GMR>Atg1* (*GMR-GAL4/Rchy1i; GMR>Atg1/+*) compared to controls (*GMR-GAL4/+* and *GMR-GAL4/*+*; GMR>Atg1/+*). **B** Quantitation of eye size represented as mm^2^ × 10^-2^ ± SD (Two-way ANOVA with Uncorrected Fisher’s LSD test). **C** Quantitation of aberrant pigment area represented as % aberrant pigment area ± SD (Two-way ANOVA with Uncorrected Fisher’s LSD test). Scale bar = 125 μm. ns non-significant. *p* < 0.05 was considered statistically significant.

To ascertain the broader role of RCHY1 in autophagy, we tested whether *Rchy1* genetically interacted with the autophagy initiation complex gene, *Atg1*. Overexpression of *Atg1* in the adult *Drosophila* eye induces autophagy hyperactivation that leads to apoptotic cell death [20]. This is characterized by reduced eye size, disruption to ommatidia arraying, and pigment loss, culminating in a rough eye phenotype [15, 20]. We utilised *GMR-GAL4* to perform *Rchy1* KD under normal (*GMR*) and *Atg1* overexpression (*GMR>Atg1*) conditions (Fig. 4A). *Rchy1* KD led to a significant decrease in eye size when *Atg1* was overexpressed compared to *Rchy1* KD under *GMR* only. However, these eyes were significantly larger than control eyes under *GMR>Atg1*, indicating rescue of eye size (Fig. 4B). We also analysed pigment loss and observed that when *Atg1* was overexpressed, *Rchy1* KD did not cause eye pigment loss to the same extent as control eyes (Fig. 4C). This observation suggests that rescue of the rough eye phenotype was due to the loss of *Rchy1* dampening the autophagy response induced by *Atg1* overexpression.

### RCHY1 facilitates autophagy flux in mammalian cells

**Fig. 5:**
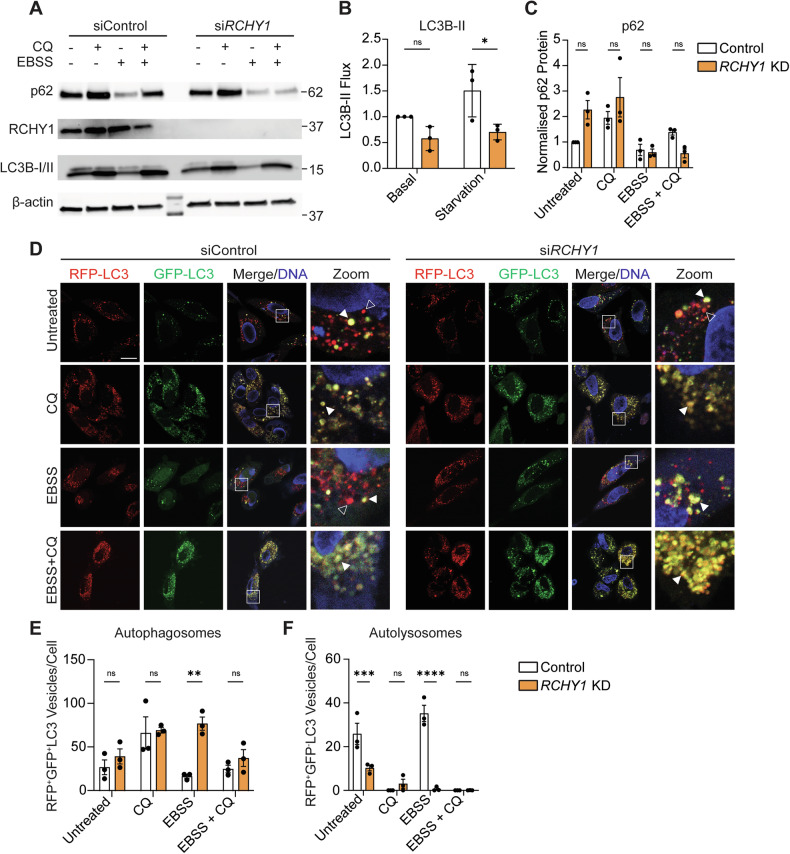
*RCHY1* depletion perturbs autophagy flux and reduces autolysosome formation. **A** Representative immunoblot for siRNA-treated *RCHY1*-depleted tandem-tagged HeLa cells stimulated with 4 h CQ and/or EBSS. Protein levels normalised to β-actin loading control. **B** Quantitation of LC3B-II flux represented as LC3B-II flux ± SD (2-way ANOVA with Bonferroni’s multiple comparisons test). **C** Quantitation of p62 levels represented as normalised p62 protein ± SD (2way ANOVA with Bonferroni’s multiple comparisons test). **D** Live-imaging of control and *RCHY1*-depleted tandem-tagged HeLa cells stimulated with 4 h CQ and/or EBSS. White boxes show zoomed regions of interest. Closed white arrows indicate autophagosomes (RFP^+^GFP^+^). Open white arrows indicate autolysosomes (RFP^+^GFP^−^). **E** Quantitation of autophagosomes represented as RFP^+^GFP^+^ vesicles/cell ± SD (2-way ANOVA with Bonferroni’s multiple comparisons test). **F** Quantitation of autolysosomes represented as RFP^+^GFP^-^ vesicles/cell ± SD (2-way ANOVA with Bonferroni’s multiple comparisons test). Scale bar (for all panels in **D**) = 10 µm. ns non-significant. *p* < 0.05 was considered statistically significant.

Many core autophagy-related proteins have conserved functions in yeast, *Drosophila*, and mammals [1]. Therefore, we hypothesised that the function of RCHY1 may be similarly conserved in autophagy in mammals. The mammalian homolog of *Drosophila* Atg8a, microtubule-associated protein 1 (MAP1) light chain 3 (LC3B) and specifically lipidated membrane-bound LC3B-II, can be used to analyse autophagy flux, or the rate at which autophagosomes are degraded by lysosomes [21, 22]. In HeLa cells, we performed siRNA-mediated knockdown (KD) of *RCHY1* (si*RCHY1*) under basal and EBSS starvation conditions and analysed LC3B-II flux by blocking lysosomal degradation using chloroquine (CQ) (Fig. 5A, B). This revealed a significant decrease in LC3B-II flux in nutrient-starved *RCHY1* KD cells (Fig. 5B). However, there was no significant difference in p62 protein levels across control and *RCHY1* KD cells (Fig. 5A, C).

The tandem-tagged autophagy flux reporter, RFP-GFP-LC3/Atg8a, can be utilised for fluorescence-based autophagy flux monitoring [23]. The probe becomes integrated into the membrane of autophagosomes following LC3B lipidation, allowing autophagosomes to be visualised by colocalisation of RFP and GFP fluorescence. Fusion of autophagosomes to lysosomes exposes the probe to the acidic lysosomal environment and quenches the pH-sensitive GFP fluorescence but retains RFP fluorescence. Therefore, the loss of GFP fluorescence can be used to detect autolysosome formation [23]. siRNA-mediated knockdown of *RCHY1* in RFP-GFP-LC3 tandem-tagged HeLa revealed significant accumulation of autophagosomes (RFP^+^GFP^+^) in EBSS-starved conditions only (Fig. 5D, E). Autolysosome (RFP^+^GFP^-^) formation was significantly attenuated in untreated and starved *RCHY1* depleted cells (Fig. 5F). This corroborates with the increase in autophagosomes observed in starved *RCHY1* depleted cells and the *Drosophila* studies that demonstrated *Rchy1* ablation in larval midguts resulted in a failure of autolysosome formation. Overall, these data support the specific role of RCHY1 in the regulation of starvation-induced autophagy in *Drosophila* and mammalian cells.

## Discussion

Previous studies have shown that in melanocytes, RCHY1 facilitates the selective autophagy of melanosomes (melanophagy) by mediating K63-linked ubiquitination of melanosomes [24]. Another possible role of RCHY1 in autophagy has been shown in non-small cell lung cancer cells, where RCHY1 is required to promote expression of autophagy-related genes during autophagosome nucleation, elongation and closure [25]. Here we report a putative new function of RCHY1 in autophagy regulation. We demonstrate that *Rchy1* deficiency results in the accumulation of autophagosomes in *Drosophila* larval midgut cells due to impaired autophagosome-lysosome fusion and that this phenotype is consistent in mammalian HeLa cells.

A key finding was the presence of large MVBs in *Rchy1*-depleted midguts that contained double-membraned autophagosomes and intact mitochondria, suggesting crosstalk between the autophagy and endocytic pathway upon *Rchy1* ablation. These individual pathways are known to intersect and form hybrid vesicles known as amphisomes that contain heterogenous materials derived from the endocytic pathway (intraluminal vesicles) and autophagy (autophagosomes and mitochondria) [19]. In mouse embryonic fibroblasts (MEFs) deficient for *Rab7* or with impaired lysosomal function, mitochondria that are usually cleared by autophagy are redirected into amphisomes which are then secreted out of the cell [26]. Our data suggest that in an effort to reduce cellular burden in *Rchy1* KD cells deficient for autophagy, autophagosomes and autophagic substrates are diverted into amphisomes that consequently accumulate. This was indicated in our Rab7/Lamp1 colocalisation analysis that revealed an increase in late endosomal compartments following *Rchy1* depletion.

As autophagy is an evolutionary-conserved pathway, we next assessed whether similar autophagy defects were observed in mammalian cells lacking *Rchy1* [12]. We found that LC3B-II flux was significantly downregulated in nutrient-starved HeLa cells upon *Rchy1* KD but not under nutrient replete conditions. This distinction is potentially due to a specific role of RCHY1 in nutrient stress conditions. Unexpectedly, we did not observe significant changes to p62 protein levels despite reduced flux. This may be due to RCHY1 interacting with a different selective autophagy receptor (SAR) in mammalian cells. A recent study has identified a link between RCHY1 and the SAR, OPTN, to mediate selective autophagy of melanosomes in B16F10 cells [24]. Because *Drosophila* lacks an ortholog of OPTN, ref(2)p may act redundantly and may be subject to dysregulation due to *Rchy1* depletion. On the other hand, OPTN may be the SAR for RCHY1 as opposed to p62 in mammalian autophagy, which could explain why p62 levels do not dramatically change in *RCHY1*-depleted cells in response to reduced autophagy flux. *RCHY1* depletion in tandem-tagged HeLa cells revealed a significant increase in autophagosomes due to impaired autolysosome formation when nutrient deprived which was consistent with our *Drosophila* findings. We also note a decrease in autolysosome formation in *RCHY1*-depleted cells in nutrient replete conditions that does not appear to affect LC3B-II flux as indicated by immunoblotting, implying that autophagosome turnover remains functional despite reduced autolysosome numbers.

Overall, our findings identify a previously unrecognized role for RCHY1 in promoting autophagosome-lysosome fusion and enhancing autophagic flux in both *Drosophila* and mammalian cells. A major limitation of this work, similar to study of RCHY1 function in melanophagy [24], is the absence of a defined RCHY1 substrate that accounts for the phenotypes we observed. Technical constraints have so far hindered our efforts to pinpoint the relevant target(s). Future studies aimed at substrate identification will therefore be essential to fully elucidate the mechanism by which RCHY1 regulates autophagosome-lysosome fusion.

## Methods

### Drosophila transgenic lines, maintenance and husbandry

The midgut driver transgenic line, *Mex-Gal4*, was provided by Richard Burke (Monash University, VIC, Australia). The following lines were obtained from the Bloomington *Drosophila* Stock Centre (Bloomington, IN, USA): *w*^*1118*^ (3605), *GMR-GAL4* (1104), *UAS-eGFP:Atg5* (BL59848), *UAS-GFP:LAMP1* (42714), *UAS-Rchy1* RNAi (58099). *UAS-Atg1* RNAi was obtained from the Vienna *Drosophila* RNAi Center. The *p-mCherry-Atg8a* transgenic line was made in-house [27]. The transgenic line used to generate mosaic clones, *hsFLP; p-mCherry-Atg8a; Act*>*CD2*>*GAL4, UAS-nlsGFP/TM6B*, was provided by Eric Baehrecke (UMass Chan Medical School, MA, USA). The *Mex-GAL4, UAS-GFP-ref(2)P/CyOactGFP; +/TM6B or TM2* was obtained from Helena Richardson (La Trobe University, VIC, Australia). *Drosophila* stocks were maintained at 18 °C, and breeding crosses were performed at 25 °C. *Drosophila* were raised on standard *Drosophila* media (18.75% compressed yeast, 10% treacle, 10% polenta, 2.5% tegosept [10% parahydroxybenzoate in ethanol], 1.5% acid mix [47% proionic acid, 4.7% orthophosphoric acid] and 1% w/v agar).

### Quantitative real time polymerase chain reaction (qRT-PCR)

Adult flies were collected one day post-eclosion, frozen at −20 °C and homogenised in TRIzol® reagent (Life Technologies, Carlsbad, CA) according to the manufacturer’s protocol. High Capacity cDNA Reverse Transcription Kit (Applied Biosciences) was utilised to create cDNA according to the manufacturer’s protocol. KAPA SYBR® FAST was used to perform qRT-PCR on a Rotor-Gene Q (Qiagen, Valencia, CA, USA) with the operating software, Rotor-Gene, according to the manufacturer’s protocol. Each reaction was performed in triplicate with three flies per sample. RNA expression was normalised to the housekeeping gene, *rp49* (beta-actin in humans) using Rotor-Gene Q 2.3.4 (Build 3) software. Primers utilised are listed below:

*Rp49* forward (5’–3’): CCAGTCGGATCGATATGCTAA

*Rp49* reverse (5’–3’): TTAGCATATCGATCCGACTGG

*Rchy1* forward (5’–3’): TGTGCAACATGTGCCTACCT

*Rchy1* reverse (5’–3’): GTATGCGCGAAGTGTGGATG

### Larval staging and midgut morphology analysis

Larvae were raised on *Drosophila* media supplemented with 0.05% bromophenol blue (Sigma-Aldrich, B6131). Wandering third-instar larvae were placed on moist Whatmann paper and gut clearance was visualised by the excretion of bromophenol blue from the gut [28, 29]. *Drosophila* larvae collected at appropriate developmental timepoints were dissected in 1×PBS and midguts were fixed in 4% paraformaldehyde. Midguts were imaged using a stereozoom microscope (Olympus, Tokyo, Japan). Gastric caeca size was calculated using the magnetic lasso tool and histogram function in Photoshop (Adobe, San Jose, CA, USA) to determine gastric caeca area in pixels, and biological measurements were derived by multiplying pixel values by pixel dimension area of images.

### Live imaging of Drosophila midguts

Appropriately-staged animals were dissected in 1xPBS supplemented with Hoechst 33342 (2 μg/ml, Sigma-Aldrich) and imaged immediately on a Carl Zeiss LSM 800 Axio Observer 7 laser scanning confocal microscope (Carl Zeiss Microscopy, Jena, Germany). For LysoTracker imaging, midguts were dissected in 1×PBS with Hoechst 33342 and 1 μM LysoTracker Red DND-99 (1:1000; Invitrogen Molecular Probes, L7528), washed for 5 min in 1×PBS, and imaged without fixation. Quantitation of puncta larger than 10 pixels was performed using ImageJ (Bethesda, MD, USA).

### Immunostaining of Drosophila midguts

*Drosophila* larvae were dissected in 1×PBS and larval midguts were fixed in 4% paraformaldehyde for 45 min at room temperature. Midguts were then washed in 3 × 5 min (PBS + 0.1% Tween-20) and 1 × 10 min in 1×PBTx (PBS + 0.1% Triton-X) and blocked in 1×PBTx + 1% BSA for 1 h at room temperature. Samples were incubated overnight in primary antibodies diluted in 1xPBTx. Primary antibodies used were rabbit anti-GABARAP (1:200, Abcam, ab109364), goat anti-GFP (1:500, Rockford, 600-101-215), mouse anti-RAB7 (1:10, Developmental Studies Hybridoma Bank, NA), rat anti-LAMP1 (1:200, BD Pharmingen, 09671D), rabbit anti-Ref(2)p (1:500, Abcam, ab178440). The following day, midguts were washed in 4 × 30 min 1×PBTx and incubated with secondary antibodies diluted in 1×PBTx for 1 h at room temperature. Secondary antibodies utilised in this study include anti-goat Alexa-FLUOR 488, anti-rabbit Alexa-FLUOR 555 and anti-mouse Alexa-FLUOR 555 (1:200, Molecular Probes, Eugene, CA, USA). Samples were washed 3 x 10 min in 1xPBTx, stained with Hoechst 33342 (2 µg/µL) for 1 min, washed in 3 x 10 min in 1×PBTx and mounted onto glass slides. Imaging was performed on a Zeiss LSM 800 confocal microscope (Carl Zeiss Microscopy, Jena, Germany). Atg8a and Lamp1 puncta with size greater than 0.1 µm^2^ were quantitated using ImageJ (Bethesda, MD, USA). Colocalised Atg8a/Lamp1 puncta were quantitated using the “image calculator” and “AND” function [15].

### Drosophila eye morphology analysis

Adult male flies were collected from breeding crosses performed at 25 °C one day post-eclosion and frozen at −20 °C. Flies were then mounted onto glass slides using nail varnish and imaged on a stereozoom microscope (Olympus, Tokyo, Japan) using 4× optical zoom. Eye size and aberrant pigment area were measured using the freehand selection tool and “measure” function, with biological scale measurements established on ImageJ (Bethesda, MD, USA) by imaging a haemocytometer grid under 4x optical zoom.

### Transmission electron microscopy

Midguts were dissected from appropriately-staged larvae in 1×PBS and fixed in 4% paraformaldehyde + 2.5% glutaraldehyde + 4% sucrose in PBS (pH 7.2) for 30 min at room temperature. Samples were then washed 2 × 5 min in 4% sucrose in PBS and post-fixed in 2% osmium tetroxide for 1 h. Midguts were dehydrated in 70% ethanol for 2 × 15 min, 90% ethanol for 2 × 15 min, 100% ethanol for 3 × 15 min, and propylene oxide for 15 min. Midgut samples were infiltrated with a 1:1 mixture of propylene oxide and epoxy resin for 1 h and 100% resin overnight. The following day, midguts were infiltrated with 100% resin for 1 h Samples were then embedded into individual resin blocks and polymerised at 60 °C for 48 h. 70 nm thick sections were cut and placed onto mesh copper grids and stained with 4% uranyl acetate for 8 min before imaging on Tecnai G2 Spirit TEM (Adelaide Microscopy).

### Cell culture and transfection

RFP-GFP-LC3 tandem-tagged HeLa cells were grown in Dulbecco’s Modified Eagle’s Medium (DMEM) supplemented with 10% foetal bovine serum, 2 mM L-glutamine, 0.5 M HEPES and 100 U/mL penicillin/streptomycin at 37 °C with 5% CO_2_. Cells were split when 70–80% confluency was achieved (approximately every 3 days).

2 × 10^5^ or 7 × 10^5^ cells were plated in 6-well plates (on coverslips for imaging) or D60 dishes, respectively, and grown for 24 h at 37 °C with 5% CO_2_. RNA-lipid complexes were prepared using 30 nM siRNA and Lipofectamine® RNAiMAX Reagent according to the manufacturer’s protocol (Thermo Fisher Scientific). Sequences for siRNA are as below:

Control (5’–3’): UUCUCCGAACGUGUCACGUTT

*RCHY1* (5’–3’): CUAGAUCGCUUUAAAGUGA

24 h following transfection, cells were washed in 1xPBS and incubated in 200 μM chloroquine (CQ) and/or Earle’s Balanced Salt Solution (EBSS) for 4 h before live-imaging or immunoblotting.

### Live imaging of cells

Following siRNA-mediated knockdown and autophagy treatments, cells were washed in 1×PBS, fixed with 4% paraformaldehyde for 3 min, stained with Hoechst 33342 diluted in PBS and mounted onto glass slides for immediate confocal imaging. Puncta > 0.1 µm^2^ were quantitated using ImageJ (Bethesda, MD, USA).

### Immunoblotting

Following siRNA-mediated knockdown and autophagy treatments, cells were trypsinised and lysed in RIPA Buffer (50 mM Tris-HCl [pH 7.4], 150 mM NaCl, 1% IGEPAL CA-630, 0.5% sodium deoxycholate, 0.1% SDS, 1 mM EDTA) containing 2% SDS and 100X HALT protease (ThermoFisher). Lysates were manually sonicated using a high-gauge hypodermic syringe and centrifuged at 16,100 × *g* for 10 min at 4 °C. Proteins were separated by electrophoresis using 4–20% precast polyacrylamide gels (Bio-Rad, USA) and transferred onto a polyvinylidene fluoride (PVDF) membrane using a Trans-Blot Turbo Transfer System (Bio-Rad, USA). Membranes were blocked in 5% skim milk in TBS-T for 1 h at room temperature and immunoblotted with primary antibodies diluted in 5% skim milk in TBS-T overnight at 4 °C. Primary antibodies utilised include rabbit anti-RCHY1 (1:1000, Abcam, ab189907), mouse anti-SQSTM1 (1:1000, Abcam, ab9126), mouse anti-β-actin (1:10,000, Sigma, AM4302), rabbit anti-LC3B (1:1000, Cell Signalling Technology, 3868). The following day, blots were washed 3 × 15 min in 1 × TBS-T and incubated with secondary antibodies diluted in 5% skim milk in TBS-T. Secondary antibodies used include anti-rabbit HRP (1:2000, Millipore), anti-mouse HRP (1:2000, Millipore) and anti-mouse Cy5 (1:2000, GE Healthcare). HRP and Cy5 signals were detected on a ChemiDoc Touch Imager (BioRad). Quantitation of immunoblot bands was performed on Image Lab Software (BioRad) and bands were normalised to β-actin.

### Confocal microscopy

Confocal images were captured on a Carl Zeiss LSM 800 Axio Observer 7 laser scanning confocal microscope with 405 nm (5 mW), 488 nm (10 mW), 561 nm (10 mW) and 640 nm (5 mW) lasers. PlanApo 40×/1.3 or 63×/1.4 Oil DIC objectives (Carl Zeiss Microscopy, Jena, Germany) were utilised. Zen 2011 (Black Edition) software was used to capture images at 40x and Airyscan detector was used to capture images at 63× [30]. Zen 3.4 (Blue Edition) was used to batch process images.

### Statistical analyses

All statistical analyses were performed using GraphPad Prism version 10.0.0 for Windows, GraphPad Software (Boston, Massachusetts USA). The statistical test/s used to analyse data sets are explicitly stated in figure legends with ns non-significant, *p < 0.05, **p < 0.01, ***p < 0.001 and ****p < 0.0001.

## Supplementary information

Supplementary Data

Uncropped Immunoblots

## Data Availability

All data analysed within this study are included either in the manuscript or in the Supplementary Materials. Any additional data relating to the work presented here will be made available on reasonable request.

## References

[1] Yamamoto H, Zhang S, Mizushima N. Autophagy genes in biology and disease. Nat Rev Genet. 2023;24:382–400. 36635405 10.1038/s41576-022-00562-w PMC9838376

[2] Farré J-C, Subramani S. Mechanistic insights into selective autophagy pathways: lessons from yeast. Nat Rev Mol Cell Biol. 2016;17:537–52. 27381245 10.1038/nrm.2016.74 PMC5549613

[3] Galluzzi L, Pietrocola F, Bravo-San Pedro JM, Amaravadi RK, Baehrecke EH, Cecconi F, et al. Autophagy in malignant transformation and cancer progression. EMBO J. 2015;34:856–80. 25712477 10.15252/embj.201490784 PMC4388596

[4] Hewitt G, Korolchuk VI. Repair, reuse, recycle: the expanding role of autophagy in Genome Maintenance. Trends Cell Biol. 2017;27:340–51. 28011061 10.1016/j.tcb.2016.11.011

[5] Klionsky DJ, Petroni G, Amaravadi RK, Baehrecke EH, Ballabio A, Boya P, et al. Autophagy in major human diseases. EMBO J. 2021;40:e108863. 34459017 10.15252/embj.2021108863 PMC8488577

[6] Pohl C, Dikic I. Cellular quality control by the ubiquitination-proteasome system and autophagy. Science. 2019;366:818–22. 31727826 10.1126/science.aax3769

[7] Shi C-S, Kehrl JH. TRAF6 and A20 regulate lysine 63-linked ubiquitination of Beclin-1 to control TLR4-induced autophagy. Science Signaling. 2010;3:ra42. 20501938 10.1126/scisignal.2000751 PMC6335036

[8] Nazio F, Strappazzon F, Antonioli M, Bielli P, Cianfanelli V, Bordi M, et al. mTOR inhibits autophagy by controlling ULK1 ubiquitylation, self-association and function through AMBRA1 and TRAF6. Nat Cell Biol. 2013;15:406–16. 23524951 10.1038/ncb2708

[9] Xia P, Wang S, Huang G, Du Y, Zhu P, Li M, et al. RNF2 is recruited by WASH to ubiquitinate AMBRA1 leading to downregulation of autophagy. Cell Res. 2014;24:943–58. 24980959 10.1038/cr.2014.85 PMC4123297

[10] Feng X, Jia Y, Zhang Y, Ma F, Zhu Y, Hong X, et al. Ubiquitination of UVRAG by SMURF1 promotes autophagosome maturation and inhibits hepatocellular carcinoma growth. Autophagy. 2019;15:1130–49. 30686098 10.1080/15548627.2019.1570063 PMC6613838

[11] Nazio F, Carinci M, Valacca C, Bielli P, Strappazzon F, Antonioli M, et al. Fine-tuning of ULK1 mRNA and protein levels is required for autophagy oscillation. J Cell Biol. 2016;215:841–56. 27932573 10.1083/jcb.201605089 PMC5166502

[12] Umargamwala R, Manning J, Dorstyn L, Denton D, Kumar S. Understanding developmental cell death using Drosophila as a model system. Cells. 2024;13:347. 38391960 10.3390/cells13040347 PMC10886741

[13] Denton D, Shravage B, Simin R, Mills K, Berry DL, Baehrecke EH, et al. Autophagy, not apoptosis, is essential for midgut cell death in Drosophila. Curr Biol. 2009;19:1741–46. 19818615 10.1016/j.cub.2009.08.042 PMC2783269

[14] Denton D, Xu T, Dayan S, Nicolson S, Kumar S. Dpp regulates autophagy-dependent midgut removal and signals to block ecdysone production. Cell Death Differ. 2019;26:763–78. 29959404 10.1038/s41418-018-0154-z PMC6460390

[15] Umargamwala R, Nicolson S, Manning J, Carosi JM, Kumar S, Denton D. Identification of new candidates regulating autophagy-dependent midgut degradation in Drosophila melanogaster. Cell Death Discovery. 2025;11:181. 40240351 10.1038/s41420-025-02474-0 PMC12003636

[16] Denton D, Kumar S. Autophagy-dependent cell death. Cell Death Differ. 2019;26:605–16. 30568239 10.1038/s41418-018-0252-y PMC6460387

[17] Chang T-K, Shravage BV, Hayes SD, Powers CM, Simin RT, Harper JW, et al. Uba1 functions in Atg7- and Atg3-independent autophagy. Nat Cell Biol. 2013;15:1067–78. 23873149 10.1038/ncb2804 PMC3762904

[18] Clague MJ, Urbé S. Multivesicular bodies. Curr Biol. 2008;18:R402–04. 18492464 10.1016/j.cub.2008.02.068

[19] Ganesan D, Cai Q. Understanding amphisomes. Biochem J. 2021;478:1959–76. 34047789 10.1042/BCJ20200917 PMC8935502

[20] Scott RC, Juhász G, Neufeld TP. Direct induction of autophagy by Atg1 inhibits cell growth and induces apoptotic cell death. Current Biology. 2007;17:1–11. 17208179 10.1016/j.cub.2006.10.053 PMC1865528

[21] Loos B, du Toit A, Hofmeyr JH. Defining and measuring autophagosome flux—concept and reality. Autophagy. 2014;10:2087–96. 25484088 10.4161/15548627.2014.973338 PMC4502790

[22] Yoshii SR, Mizushima N. Monitoring and measuring autophagy. Int J Mol Sci. 2017;18:1865. 10.3390/ijms18091865 PMC5618514 28846632

[23] Kimura S, Noda T, Yoshimori T. Dissection of the autophagosome maturation process by a novel reporter protein, tandem fluorescent-tagged LC3. Autophagy. 2007;3:452–60. 17534139 10.4161/auto.4451

[24] Lee KW, Ryu KJ, Kim M, Lim S, Kim J, Kim JY, et al. RCHY1 and OPTN are required for melanophagy, selective autophagy of melanosomes. Proc Natl Acad Sci USA 2024;121:e2318039121. 38536750 10.1073/pnas.2318039121 PMC10998605

[25] Fedorova O, Gudovich A, Daks A, Baidyuk E, Shuvalov O, Petukhov A, et al. Regulation of autophagy flux by E3 ubiquitin ligase Pirh2 in lung cancer. Biochem Biophys Res Commun. 2021;563:119–25. 34090148 10.1016/j.bbrc.2021.05.024

[26] Liang W, Sagar S, Ravindran R, Najor RH, Quiles JM, Chi L, et al. Mitochondria are secreted in extracellular vesicles when lysosomal function is impaired. Nat Commun. 2023;14:5031. 37596294 10.1038/s41467-023-40680-5 PMC10439183

[27] Denton D, Chang TK, Nicolson S, Shravage B, Simin R, Baehrecke EH, et al. Relationship between growth arrest and autophagy in midgut programmed cell death in Drosophila. Cell Death Differ. 2012;19:1299–307. 22555456 10.1038/cdd.2012.43 PMC3392632

[28] Maroni G, Stamey S. Use of blue food to select synchronous, late third instar larvae. Dros Inf Serv. 1983;59:142–43.

[29] Bainbridge SP, Bownes M. Staging the metamorphosis of Drosophila melanogaster. Development. 1981;66:57–80. 6802923

[30] Huff J. Application Notes: The Airyscan detector from ZEISS: confocal imaging with improved signal-to-noise ratio and super-resolution. Nat Methods. 2015;12:i–ii.

