# Supplementary material for: Ubiquitin ligase RCHY1 regulates autophagosome-lysosome fusion
Source: Cell Death Discov. 2026 Apr 15;12:247. doi: 10.1038/s41420-026-03088-w (PMC13194709; doi:10.1038/s41420-026-03088-w)
Supplement: Supplementary file 1 — Supplementary Data [file 41420_2026_3088_MOESM1_ESM.docx]

**
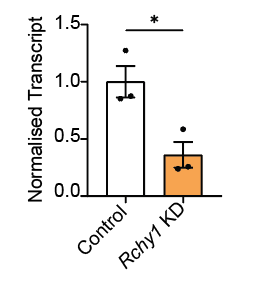
**

**Supplementary Fig. 1:** *Rchy1* KD efficiency in *Drosophila*. Relative mRNA levels of RCHY1 in control (*Act-GAL4/+*) and *Rchy1* KD (*Act-GAL4/Rchy1i*) adult male flies one day after eclosion. Expression levels were calculated relative to the housekeeping gene, *rp49*, and normalised to control. Each reaction was performed in triplicate with three flies per sample. Data are represented as mean normalised transcript ± SEM (unpaired t-test). *p* < 0.05 was considered statistically significant.


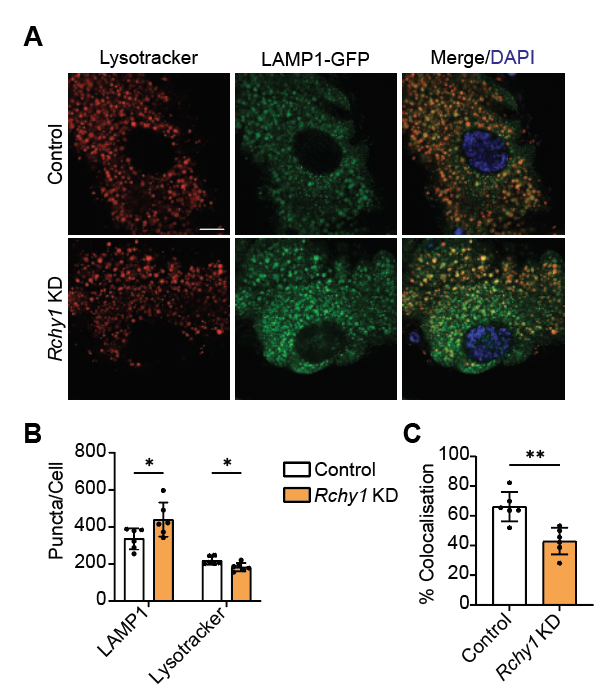
**Supplementary Fig. 2:** *Rchy1* KD results in fewer acidified LAMP1-positive vesicles in larval midgut cells. (**A**) Live-imaging of control (*Mex-GAL4, UAS-LAMP1-GFP/+*) and *Rchy1* KD (*Mex-GAL4, UAS-LAMP1-GFP/UASRchy1i*) larval midguts at -4 h RPF expressing LAMP1-GFP (green) co-stained with LysoTracker (red). (**B**) Quantitation of LAMP1- and LysoTracker-positive puncta represented as puncta/cell ± SD (unpaired t-test). (**C**) Quantitation of colocalised LAMP1- and LysoTracker-positive vesicles represented as puncta/cell ± SD (unpaired t-test). Scale bar = 10 µm. *p* < 0.05 was considered statistically significant.
