# Supplementary figures and images for: Ubiquitin ligase RCHY1 regulates autophagosome-lysosome fusion
Source: Cell Death Discov. 2026 Apr 15;12:247. doi: 10.1038/s41420-026-03088-w (PMC13194709; doi:10.1038/s41420-026-03088-w)

**Fig. 5A – Uncropped blots**

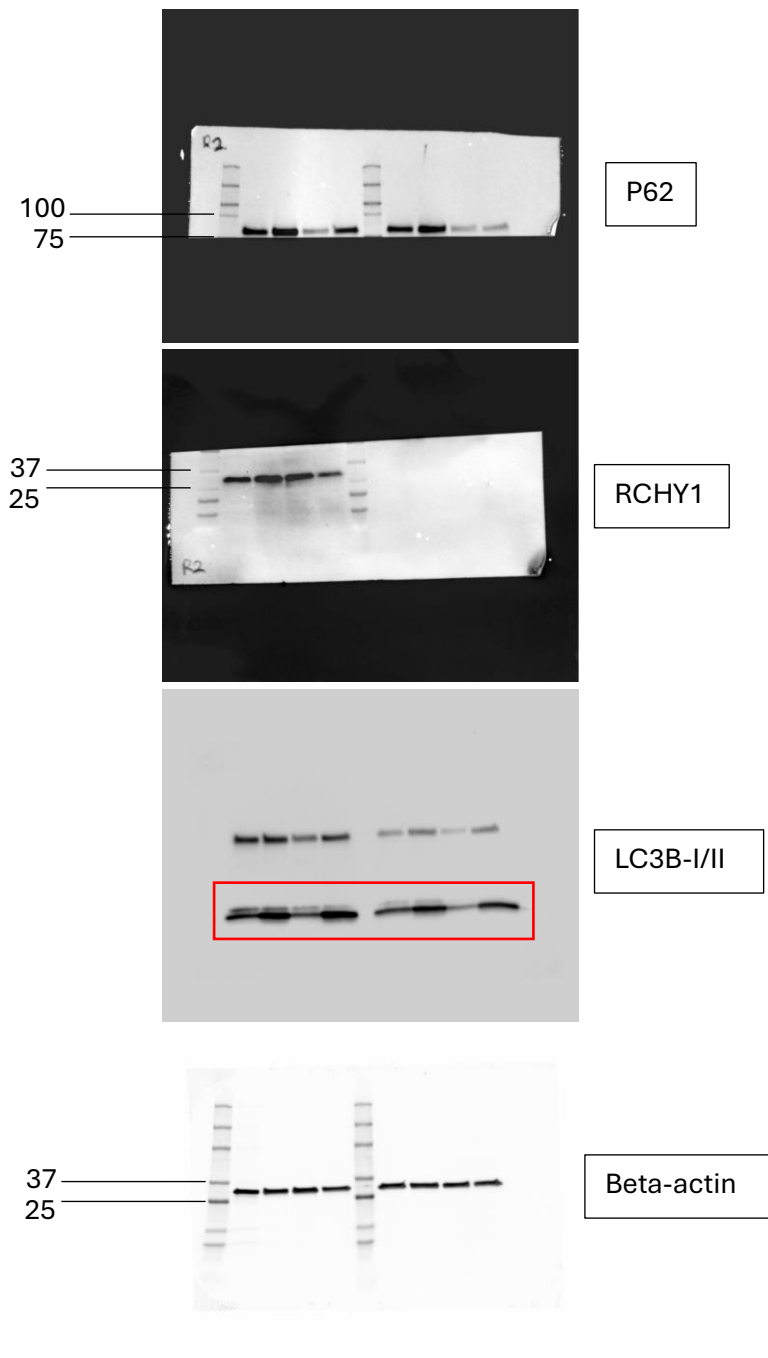

Supplement: Supplementary file 2 — Uncropped Immunoblots [file 41420_2026_3088_MOESM2_ESM.pdf]
